# A mass-density model can account for the size-weight illusion

**DOI:** 10.1371/journal.pone.0190624

**Published:** 2018-02-15

**Authors:** Christian Wolf, Wouter M. Bergmann Tiest, Knut Drewing

**Affiliations:** 1 Experimental Psychology, Justus-Liebig-University Giessen, Giessen, Germany; 2 Experimental and Biological Psychology, Philipps-University Marburg, Marburg, Germany; 3 School of Communication, Media & Information Technology, Rotterdam University of Applied Sciences, Rotterdam, the Netherlands; University of Exeter, UNITED KINGDOM

## Abstract

When judging the heaviness of two objects with equal mass, people perceive the smaller and denser of the two as being heavier. Despite the large number of theories, covering bottom-up and top-down approaches, none of them can fully account for all aspects of this size-weight illusion and thus for human heaviness perception. Here we propose a new maximum-likelihood estimation model which describes the illusion as the weighted average of two heaviness estimates with correlated noise: One estimate derived from the object’s mass, and the other from the object’s density, with estimates’ weights based on their relative reliabilities. While information about mass can directly be perceived, information about density will in some cases first have to be derived from mass and volume. However, according to our model at the crucial perceptual level, heaviness judgments will be biased by the objects’ density, not by its size. In two magnitude estimation experiments, we tested model predictions for the visual and the haptic size-weight illusion. Participants lifted objects which varied in mass and density. We additionally varied the reliability of the density estimate by varying the quality of either visual (Experiment 1) or haptic (Experiment 2) volume information. As predicted, with increasing quality of volume information, heaviness judgments were increasingly biased towards the object’s density: Objects of the same density were perceived as more similar and big objects were perceived as increasingly lighter than small (denser) objects of the same mass. This perceived difference increased with an increasing difference in density. In an additional two-alternative forced choice heaviness experiment, we replicated that the illusion strength increased with the quality of volume information (Experiment 3). Overall, the results highly corroborate our model, which seems promising as a starting point for a unifying framework for the size-weight illusion and human heaviness perception.

## Introduction

When we lift two objects of equal mass, we perceive the smaller and denser of the two as being heavier. This size-weight illusion (SWI) was first described by Charpentier [1,2]. It occurs consistently across participants [3] and persists after participants have been told that objects are equal in mass [4]. It does not depend on whether two objects are lifted consecutively or with both hands at the same time [5]. However, there is some controversy about how to explain the illusion.

One line of research tried to address the illusion from a top-down perspective. Here, the illusion is thought to arise because either cognitive heaviness expectations or expectations of the motor system are not met [6,7]. When people lift two stimuli of equal mass, the larger of the two is lifted with a higher force [8]. As a consequence, the larger object might be lifted with too much and the smaller one with too little force. Early accounts suggested that the mismatch between required and applied force gives rise to the perceptual illusion. But as has meanwhile been shown [9–12], people rapidly adapt their lifting forces towards the required masses while the perceptual illusion remains constant, disproving that the illusion is due to this mismatch in forces.

Instead many recent authors emphasize a role for cognitive heaviness expectations [13–16]: In most objects we encounter every day, size and mass are positively correlated [17], i.e. larger objects on average are heavier than smaller ones. This is especially true when large and small objects are of the same material (i.e. of constant density). The SWI is then explained by combining present sensory evidence on an object’s mass with prior knowledge about how size and mass relate in the physical world, i.e. with a prior on how heavy the object is expected to be, given its size. [6,10,11]. The combination of sensory evidence with prior expectation is generally modelled within a Bayesian framework. Bayesian models or models using maximum-likelihood estimation (MLE) rely on the same underlying principle: Prior or other sensory information pointing to a certain value of a property will shift perception towards that value. According to this principle, prior expectations should bias perception towards the expectation, i.e. if the illusion were caused by a size-weight prior (“larger objects are heavier”), the larger of two equally-weighted objects should be perceived as heavier. But in the SWI larger objects are perceived as lighter, and the perceptual illusion was modeled as an "anti-Bayesian" contrast effect [10], in which current sensory information is contrasted instead of being integrated with prior expectations. It can be considered unlikely that heaviness perception behaves anti-Bayesian, given that information integration in perception has been successfully described by Bayesian or MLE models in so many previous cases, regarding, e.g. multiple sensory cues [18–21], sensory information over time [22,23], the integration of multiple spatial reference frames [24,25] as well as perception across eye movements [26,27]. Thus, assuming that the illusion is caused by a size-weight prior or a direct use of size information is not in line with the principles that nowadays provide the best description of our perceptual system.

The illusion is strongest when participants obtain haptic information on the object’s size, weaker but also present when only visual size information is available and does not appear when participants are neither provided with visual nor haptic size information [28–30], e.g. when they are blindfolded and lift objects using a string or handle. The simultaneous availability of visual and haptic size information does not yield a stronger illusion compared to the pure haptic size-weight illusion [28,30,31]. Studies that manipulated the time point, when visual size information was presented, provide further evidence against the role of prior expectations: Masin and Crestoni [29] let participants lift objects either without size information, or with visual size information before or during lifting. The illusion was only observed when size information was presented at the same time as weight information. Size information prior to lifting, which can be expected to induce cognitive expectations on the object’s weight, did not induce a size-weight illusion. In an experiment by Buckingham and Goodale [11], participants repeatedly lifted the same cube but were either shown a small or a large cube shortly before lifting. In this study, people reported perceiving the cube as heavier after having seen the small cube, but the strength of the illusion was only a fraction (around one half) of the illusion with visual size information present during lifting. Thus, if expectations contribute to the SWI, their contribution is comparatively weak [11,13]. Consequently, prior size expectations cannot account for the full size-weight illusion.

A different line of research has focused on bottom-up mechanisms that might give rise to the SWI and on how we internally integrate the presently available information about mass and size in order to form a common percept of heaviness [32–35]. Anderson [33] assumed a weighted average of two independent heaviness estimates, one derived from mass and the other one derived from size, with the latter one being subtracted from the first. One prediction of this model is that the perceived difference of two objects of equal mass depends on size only. However, because the influence of size on perceived heaviness also depends on the objects' mass [34–36], his integration model cannot explain why the illusion strength depends on both parameters, mass and size. Other bottom-up explanations suggest influences of lifting kinematics and inertial characteristics on perceived heaviness [37,38]. Whereas corresponding studies nicely show the influence of different types of haptic information, they cannot explain why the illusion occurs when size information is provided by the visual modality only.

The fact that the illusion varies as a function of both parameters, mass and size, suggests that the illusion might be related to density [39,40] (cited from [30,41]), because mass and size are related by an object’s density, i.e. mass divided by volume. In the case of two equally-weighted stimuli of different size, the smaller one is denser than the larger one. Whereas mass can directly be perceived by the haptic and size by the haptic and/or visual modality, information about an object’s density can often only be obtained by combining information about mass and size. Recent studies show that the brain computes density and that this information is used for heaviness perception [17,42]. The usage of density information is in line with the common principle of Bayesian and MLE statistics, because higher density values should bias perception towards higher heaviness estimates. Thus, it correctly predicts that denser (i.e. smaller) objects are perceived as heavier than less dense (i.e. larger) objects of the same mass. For any constant mass, perceived heaviness increases with the object’s physical density [34,35,40]. Thus an object’s density rather than size might be the second component, next to its mass, which is considered in the judgment of heaviness and which biases our perception in terms of the size-weight illusion. Calculating and storing object information about density might be efficient for the motor system when lifting objects, because combining the memorized density with a visual size analysis [43,44] would be sufficient to prepare an appropriate motor command for a whole family of objects made from the same material.

According to now well-established models of information integration in perception [19,45], the brain integrates all signals available for a property in order to maximize the reliability of the final percept. Signal-specific estimates ${\hat{s}}_{i}$ for that property are derived from each signal *s_i_*. Then all estimates combine into a single percept by weighted averaging:

$$\hat{s}=\sum_{i}w_{i}{\hat{s}}_{i}\;with\;\sum_{i}w_{i}=1;\;w_{i}\in [0,1]$$ (1)

For every signal *s*_*i*_ its reliability, *r*_*i*_, is given by the inverse of the estimate’s variance (r_i_ = σ_i_^-2^). When cues (e.g. from different modalities) are integrated, it is generally assumed that the estimates’ noises are independent. In that case, optimal weights are given by their relative reliability:

$$w_{i}=\frac{r_{i}}{\sum_{i}r_{i}}$$ (2)

If optimal weights are chosen, the reliability of the combined percept is maximized and equals the sum of the individual reliabilities:

$$r_{tot}=\sum_{i}r_{i}$$ (3)

Here, we propose a model [46] which describes perceived heaviness as a function of mass and density, *ĥ(m*,*ρ)*. It is formed as a weighted average of two heaviness estimates with correlated errors [20], one estimate derived from mass, *ĥ*_*m*_*(m)*, and the other derived from density, *ĥ*_*ρ*_*(ρ)*. The model assumes that a density estimate is first derived from mass and size information and subsequently integrated with the mass estimate. Thus, at the final perceptual level, heaviness perception is influenced by the object’s density–not by its size. The model can be described by:

$$\hat{h}(m,\rho )=(1-w_{\rho }){\hat{h}}_{m}(m)+w_{\rho }{\hat{h}}_{\rho }(\rho ).$$ (4)

In case of the size-weight illusion and the integration about mass and density, the estimates’ noises cannot be considered independent. Because a density estimate would have to be derived from mass and size, the density estimate would contain noise from both estimates. Consequently, the mass and the density estimate will also partly share the same noise and their errors will be positively correlated. In case of two cues with correlated errors, the optimal weight for a signal specific estimate, *w*_1_, is given by [20]:

$$w_{1}=\frac{r_{1}-\varrho \sqrt{r_{1}r_{2}}}{r_{1}+r_{2}-2\varrho \sqrt{r_{1}r_{2}}}$$ (5)

with *ϱ* denoting the error correlation. The weight for the second cue can then be set to *w*_*2*_ *= 1 –w*_*1*_. If the correlation *ϱ* is zero, Eq (5) shortens to Eq 2. If *ϱ* equals one, both cues would be assessed with an identical error and one cue would receive a weight of 0. In the correlated case, the less reliable of both cues receives a lower and the more reliable one a higher weight as compared to the uncorrelated case [20]. If optimal weights are chosen, the reliability of the combined percept is:

$$r_{int}=\frac{r_{1}+r_{2}-2\varrho \sqrt{r_{1}r_{2}}}{1-\varrho^{2}}$$ (6)

Again, if *ϱ* is zero, Eq (6) reduces to Eq (3). Eq (5) allows optimal weights to become negative (see [20] for further discourse). As long as weights are non-zero, the combined percept will be more reliable than the single cues [20]. For positive weights, the combined reliability *r*_*int*_ decreases with increasing correlation *ϱ*. If a weight is zero, the total reliability equals the reliability of the other (i.e. the more reliable) cue. As long as both cue reliabilities are not completely dissimilar (i.e. if the square-rooted ratio of the reliabilities does not fall below the noise correlation *ϱ*, [20]), even larger changes in *ϱ* will not lead to negative weights and thus only affect quantitative predictions in the ways described above.

The two estimates are supposed to describe the relationship between the physical and perceived stimulus intensity. In psychophysics, the relationship between the physical intensity of a stimulus and its perceived intensity is usually best described by Stevens’ power law [47]. We therefore model the mass and the density estimate as power functions of their underlying physical parameter:

$${\hat{h}}_{m}(m)=am^{x},\;{\hat{h}}_{\rho }(\rho )=b\rho^{y},$$ (7)

where *a*, *b*, *x*, and *y* are constants. Generally, the model predicts that (i) perceived heaviness increases independently with mass and with density. For two equally-weighted objects, (ii) the denser (i.e. the smaller) of the two is perceived as heavier and (iii) this perceptual difference increases with an increasing difference in the objects’ density. The illusion strength is reflected by the degree to which density information is weighted. In our model, the illusion strength is revealed by the density weights *w*_*ρ*_. (iv) Density weights and thus the illusion magnitude should be greater for a more reliable density estimate. (v) The overall heaviness estimate should be more reliable when density information is integrated (i.e. when people are deceived by the illusion) compared to when heaviness estimates are only based on information about mass. Additionally, (vi) the influence of the less reliable estimate and its reliability should decrease with an increasing error correlation. If the density estimate is less reliable than the mass estimate, then an increase in the error correlation would decrease the contribution of the density estimate. Here, we did not test the last two predictions because we aimed to test those predictions (i-iv) which follow both from the correlated and uncorrelated case and which are also concerned with biasing perceived heaviness (i-iv). Moreover, the error correlation would only diminish the potential effect of density (i-iii) and its reliability (iv). Even if the error correlation varies systematically with stimulus intensity or the way objects are lifted, this would only reduce the influence of density and its reliability (see Discussion of Experiment 1 & 2). Thus, predictions (i) to (iv) are testable independent of the error correlation.

To test our model, we conducted three experiments where people had to lift objects which varied in mass and density. We additionally aimed to vary the reliability of the density estimate by manipulating the quality of either visual or haptic size information. In the first two experiments we used magnitude estimation to test our model predictions (i) to (iv) on perceived heaviness. If the illusion is caused by the integration of a mass and a density estimate, then the pattern of magnitude estimates should reflect the physical pattern of object mass and density. The extent to which density information is weighted is supposed to depend on the reliability of the density estimate. In our experiments, we aimed to manipulate the reliability of the density estimate by manipulating the quality of size information. In Experiment 1, participants lifted objects on a string and thus had visual size information only. We impaired vision to different extents in order to test whether with increasing visual impairment, the illusion decreases as density information becomes less reliable. This experiment and an earlier version of the model has been described previously in conference proceedings [46]. In Experiment 2, blindfolded participants lifted objects with grips providing different qualities of haptic size information. Again, the illusion should be strongest with a grip type providing high quality haptic volume information. In a third experiment, we tested whether our model can describe perceived heaviness with a method which should be less prone to influences from higher level, cognitive processes as compared to magnitude estimation. We measured the strength of the size-weight illusion by using a two-interval forced choice (2IFC) task. For every individual, observed shifts in perceived heaviness were compared against model parameters obtained by magnitude estimation.

## Experiment 1: The visual size-weight illusion

According to our model, there are four major factors influencing the perceived heaviness of an object: its mass, its density, the relative reliabilities of both cues and the noise correlation. In these experiments, we manipulated the first three factors: Participants had to lift objects that varied in mass and density and judge their heaviness in a free magnitude estimation task [47]. All objects were lifted on a string to elicit a purely visual size-weight illusion [28]. Although at the final level, perception is biased by density information, the density estimate will first have to be derived using information about mass and size. Increasing the reliability of any of the two would also increase the reliability of the density estimate. Thus, to manipulate the reliability of the density estimate while keeping the reliability of the mass estimate constant, we additionally varied visibility in four discrete steps. Participants either had no vision, strongly impaired vision, mildly impaired vision, or full vision.

**Fig 1:**
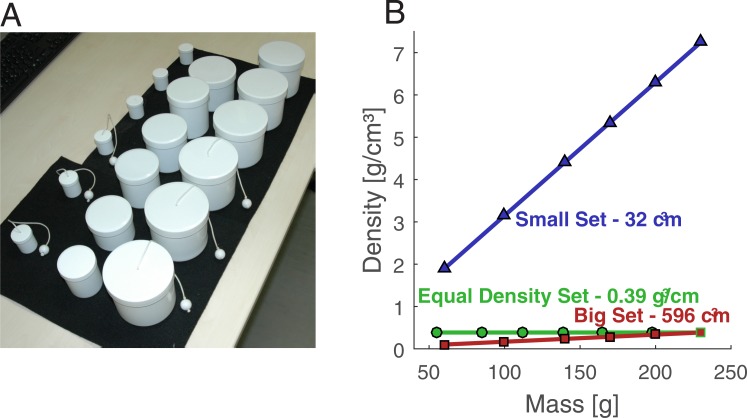
Stimuli and their physical parameters. (A) All stimuli used in Experiment 1 and 2. Screw caps were removable and could either be plain or attached with a string and a wooden bead. Small set objects are in the back, big set objects in the front row and equal-density objects in in-between. (B) Physical parameters of stimuli in (A). Small and big set objects were constant in volume and thus their density increased with increasing mass. This increase is more prominent in the small set. Objects from the equal density set were constant in density. The 230 g stimulus of the big set also had a density of 0.39 g/cm^3^ and was also included in the analysis of the equal density set.

Stimuli (Fig 1A) comprised three stimuli sets: a small set (high density), a big set (low density) and an equal-density set. The two volume sets (small and big set) contained 6 pairs of equally weighted objects. As volume was constant in these two sets, the stimuli increased in density with increasing mass (Fig 1B). Importantly, the absolute difference in density between equal-weight stimuli of the two volume sets got larger for heavier stimuli. The third set, the equal density set, contained objects of the same density. The heaviest object from the big set was also included in the analysis of the equal density set as it had the same density.

Our model predicts higher heaviness estimates with increasing mass. If no information about density is available (blindfolded condition), heaviness estimates are supposed to be a function of mass only. If density information is present (poor, medium and full vision condition), heaviness estimates are supposed to differ and increase differently for the three stimuli sets. Given the properties of our stimuli sets, our model predicts:

a) Estimates are larger for small-volume than for big-volume objects with same mass–and in-between for the equal-density set. This is the classical size-weight illusion.
b) Estimates of the volume sets increase more with mass than estimates of the equal-density set, because in the volume sets higher mass coincides with higher density.
c) The difference between heaviness estimates for small- and big-volume objects increases with mass, because the absolute difference in density also increases.
d) As a better visibility should lead to a more reliable density estimate, the differences denoted in a, b and c become more pronounced with increasing visibility.

### Methods

#### Participants

We recruited 15 students from Giessen University, aged 19 to 44 years with a mean age of 27 years (12 females; 6 left-handed according to self-report). All students received course credit for participation. Participants reported to have no known sensory or motor deficits. Their vision was normal or corrected by contact lenses. They were naïve as to the purpose of the experiment and to the size-weight illusion. For all experiments, informed written consent was obtained before testing. All experiments reported in this study were approved by the local ethics committee of FB06 at the University Giessen and conducted in accordance with the ethical standards laid down in the 1964 Declaration of Helsinki.

#### Setup and stimuli

We created 18 stimuli using cylindrical white plastic cans with a screw cap (Fig 1). The cans differed in volume, which we assessed by the method of water displacement. Each stimulus had a homogenous mass distribution over its entire volume. An exception is the 60g-stimulus with big volume, because the can alone already had a mass of close to 60 g and we distributed only some material homogeneously over this can’s inner surface. The filling materials were mixtures of iron or tungsten powder with silicone or with polyurethane foam. A string of 20 cm length and 1.3 mm diameter was attached to the center of each screw cap. To blindfold participants, they wore a sleeping mask (no vision). For the medium and poor visual condition, participants wore differently manipulated diving goggles. In one pair of goggles, we stuck a transparent colorless cling film (“d-c-fix 7”) to the glasses in order to strongly impair vision (poor vision). In the other goggles we laid 16 layers of regularly crumpled commercial wrapping film, resulting in an intermediate visual impairment (medium vision).

Participants sat in front of a table with their elbows at about the same height as the table surface. On the table was a sound-absorbing felt pad (30 × 40 cm), on which the current stimulus could be placed. Other stimuli were hidden from the participant’s view. Participants lifted the object by grasping a wooden bead (13 mm diameter) at the other end of the string. There were 6 stimuli with a volume of 596 cm^3^ and masses of 60, 100, 140, 170, 200 and 230 g (big, low-density set, densities of 0.10, 0.17, 0.24, 0.29, 0.34, and 0.39 g/cm^3^), 6 stimuli with a volume of 32 cm^3^ and the same 6 masses (small, high-density set, densities of 1.93, 3.19, 4.43, 5.36, 6.31, and 7.24 g/cm^3^), and 6 stimuli with a density of 0.39 g/cm^3^ and masses of 55, 85, 112, 139, 165 and 198 g (equal-density set). The 230 g stimulus in the big volume set had the same density of 0.39 g/cm^3^ as the equal-density stimuli and was included in the analyses of the equal-density set. For inferential statistics, we used the 60, 140 and 230g stimuli from the volume sets and matched them with equal-density stimuli that were closest in mass (55g, 139 and the 230 g stimulus also included in the big set). Masses of equal density stimuli deviated slightly from the masses in the other two sets, because the available volumes of plastic cans were limited. However, mass deviations for the stimuli used for inferential statistics were smaller than 10% (60 and 140 g for the volume sets, 55 and 139 g for the equal-density set). Throughout the remainder of the paper, we will also refer to the 55 and 139 g stimuli of the equal-density set when we speak of 60 and 140 g stimuli.

#### Design and procedure

The design comprised three within-subject variables: Mass, Density Set (big-volume, small-volume, equal-density) and Visibility (no, poor, medium and full vision). In the no vision condition participants were blindfolded. In the poor and medium vision condition they wore the strongly and intermediately impairing diving goggles, respectively. No glasses or masks were used in the full vision condition. Visibility conditions were presented in a fixed order so that Visibility increased during the experiment and no transfer of information into lower Visibility conditions could occur (see General Discussion for discourse of potential order effects). Each participant started with the no vision condition, and went on with the poor, then, the medium and, finally, the full vision condition. There were 18 stimuli belonging to the different Density Sets and Mass conditions (Fig 1). In each block of the no and full vision conditions, each of the 18 stimuli was presented once. In the poor and medium vision condition, we only presented stimuli which were also used for inferential statistics: 60, 140 and 230 g stimuli (this included the 55 and 139 g stimuli from the equal-density set). The main purpose of the two intermediate conditions (poor and medium vision condition), was to show continuity of the changes in effects from null to full vision for which a selected number of stimuli was sufficient. Moreover, including all stimuli in the poor and medium condition would have further prolonged the already lengthy experiment. Participants were instructed to judge the stimuli according to perceived heaviness in a free-modulus magnitude estimation task [47]. Judgments were made by assigning a whole number or any fraction larger than 0 to each stimulus that best described its perceived heaviness. No standard or modulus was used.

In every trial, the experimenter first silently placed a stimulus on the felt pad in front of the participant. The participant was instructed to extend his/her dominant hand at about 30 cm above the table with palm down and the experimenter placed the stimulus’ wooden bead between the participant’s thumb and index fingers. Then, the participant lifted the stimulus via bead and string and moved it up and down twice. Within the next 3 seconds the participant had to judge the stimulus’ heaviness and, afterwards, let the stimulus down to the felt pad. S/he was instructed to always move slowly in order to minimize lateral swaying of the stimulus and, thus, to minimize potential, but unwanted, haptic cues to mass distribution (40). Finally, the experimenter removed the stimulus, noted the participant’s judgment and the next trial began. In the conditions including vision, the participant was instructed to look at the stimulus throughout the trial.

After initial instructions, each participant started with a block in the no vision condition that served to practice the exact procedure as well as to establish a subjective heaviness scale. During practice each of the 18 stimuli was presented once. Practice trials were not considered for the analysis. After practice, participants were instructed to “keep the subjective scale constant” and the proper experimental trials began. Each Visibility condition comprised six blocks. A block consisted of 18 trials for the no vision and full vision condition and 8 trials in the poor and medium vision condition. Within each block, stimuli were presented in random order. Overall the experiment comprised 18 stimuli × 6 blocks × 2 conditions + 8 stimuli × 6 blocks × 2 conditions. This resulted in 312 experimental trials per participant. After the experiment, we measured the participant’s visual acuity while wearing either pair of diving goggles, thus for the poor and medium vision condition. We used an enlarged version of Landolt rings with openings in the ring in a range of 0.5 to 32 mm. Landolt rings were placed at the same location as the heaviness stimuli. The experiment lasted about 3 hours including 3 breaks of 3 minutes.

#### Data analysis

The raw data consisted of each participant’s six magnitude estimates of the heaviness for each stimulus in each Visibility condition. In a first step, each single estimate was standardized: For each participant, each estimate was divided by the participant’s geometric mean over all estimates. From these values, we calculated the individual geometric mean per condition. These individual scores were used in additional statistical analyses. Data from the 230 g stimulus in the big volume set was used both in the big volume and the equal-density set. Individual scores of the estimated heaviness were compared using a repeated-measures ANOVA with the 3 within-participant factors Mass (60, 140 and 230 g), Density Set (small set, equal-density set, big set) and Visibility (no vision, poor, medium, full vision). For the ANOVA, we matched the data from the 60, 140 and 230 g stimulus in the volume sets with the data from that stimulus in the equal-density set that is closest in mass. Thus, for the ANOVA, the 56 g and the 139 g equal-density stimuli were matched with the 60 and 140 g stimuli of the two volume sets. The statistical procedure for this experiment was identical to the procedure in Experiment 2 and 3: P-values and degrees of freedom from repeated-measures ANOVAs were corrected according to Greenhouse-Geiser when sphericity was violated. To test the direction of effects, repeated-measures ANOVAs were followed up by planned comparisons. If we compared two conditions, we used paired-sample t-tests, if we compared more than two conditions, we employed linear contrasts. All analyses were based on a-priori model predictions and our model also predicts the direction of effects. All planned comparisons were one-tailed and we did not control for multiple testing. For ANOVAs and linear contrasts, effect sizes were calculated as partial eta-squared, η_p_^2^. For t-tests, we report Cohen’s d as effect size.

Model fits (Eqs 4 & 5) were done by numerically minimizing squared errors of the predicted from the measured values: For a given set of parameters, we first computed the difference between the theoretical model predictions and the observed heaviness estimates. The sum of these squared errors was minimized within the fitting procedure. To avoid local minima, we randomly perturbed one, two and all of the parameters within one iteration. The whole procedure was repeated for 1000 iterations and the set of parameter which yielded the lowest squared residual error was taken as result of the fitting procedure.

### Results

#### Visual acuity

To make sure that our intended manipulation of Visibility was successful, we compared visual acuity in the two impaired visual conditions. Visual acuity was assessed by the minimal visual angle of the gap in the Landolt rings that participants were able to locate correctly. Participants had an average acuity of 12' (minute of arc) with the intermediate impaired goggles (range: 6'-21') and an acuity of 78' (range: 32'-139') in the poor visual condition. Thus, vision was significantly impaired in the poor compared to the medium visual condition, *t*(14) = 7.9, *p* < 0.001, *d* = 4.19. We did not measure the participants’ normal visual acuity without diving goggles, but in healthy individuals the normal visual acuity is between 1' and 2’ [48], and thus below the ranges of acuities we measured for both impaired visibility conditions.

#### Heaviness estimates

**Fig 2:**
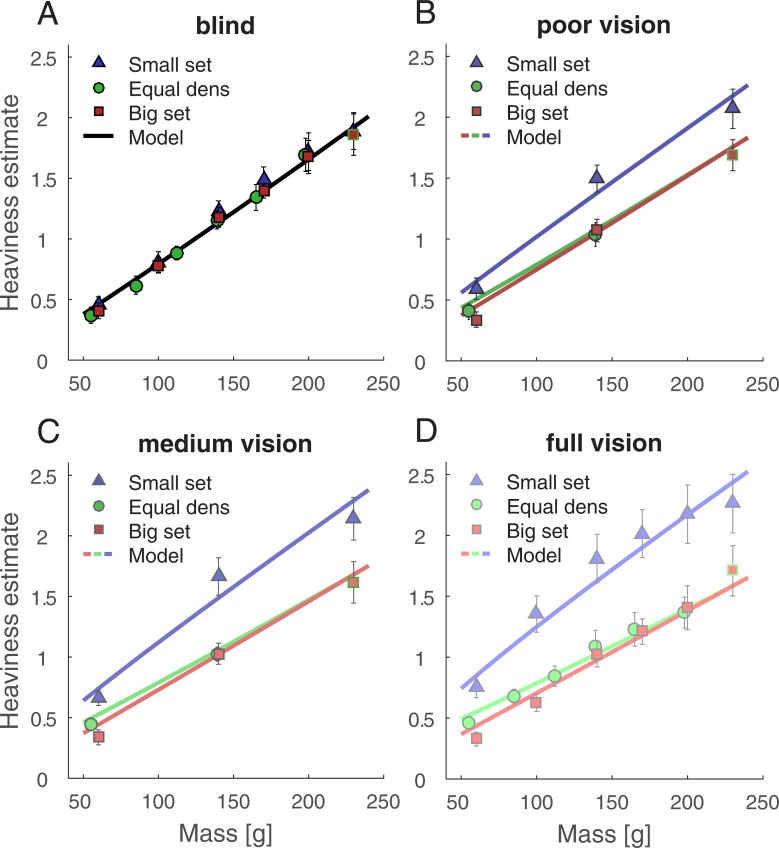
Main results of Experiment 1. Heaviness estimates and model fits for the blindfolded (A), the poor (B), the medium (C) and the full vision condition (D). Estimates are averaged over individual heaviness scores with error bars denoting 95% confidence intervals. Lines represent the model fit.

As predicted, heaviness estimates, on average, significantly increased with Mass (Fig 2, see also S1 Fig). Average heaviness estimates for the 60, 140 and 230 g stimuli were 0.47, 1.25 and 1.86. The repeated-measures ANOVA revealed a significant main effect of mass, *F*(1.19,16.8) = 236, *p* < 0.001, *η*_*p*_^*2*^ = 0.94. 140 g stimuli were judged heavier than 60 g stimuli, *t*(14) = 16.6, *p* < 0.001, *d* = 4.3, and 230 g stimuli were judged heavier than 140 g stimuli, *t*(14) = 11.9, *p* < 0.001, *d* = 3.08. Heaviness estimates also, on average, significantly differed between the Density Sets in the way predicted from the pattern of density differences: Overall, estimates were smaller for the big-volume set (1.05) than for the equal-density set (1.07) than for the small-volume set (1.42), main effect Density Set, *F*(1.11,15.5) = 139, *p* < 0.001, *η*_*p*_^*2*^ = 0.91. Small, high-density objects were judged heavier than equal-density objects, *t*(14) = 12.0, *p* < 0.001, *d* = 3.10, and equal-density objects were judged heavier than big, low-density objects, *t*(14) = 2.9, *p* = 0.007, *d* = 0.74.

Physically, the density difference between the small set and the other two stimuli sets increases with mass, whereas the density difference between equal-density and big objects decreases with mass (Fig 1B). The effect of density should thus be modulated by mass. As predicted by our model, this density pattern is reflected in a significant Mass × Density Set interaction in the heaviness estimates, *F*(2.12,29.7) = 14.8, *p* < 0.001, *η*_*p*_^*2*^ = 0.51. To further examine the direction of this interaction, we used linear contrasts. First, we computed pairwise differences between equally-weighted objects of two density sets (averaged across all Visibility conditions) and then computed a linear contrast over all Mass conditions. As revealed by linear contrasts and a t-test, the difference in heaviness estimates increased between the small and the equal-density set with increasing mass, *F*(1,14) = 26.4, *p* < 0.001, *η*_*p*_^*2*^ = 0.65 (60 g: 0.19, 140 g: 0.47, 230 g: 0.37), it increased between the small and the big set, *F*(1,14) = 9.0, *p* = 0.005, *η*_*p*_^*2*^ = 0.39 (60 g: 0.26, 140 g: 0.47, 230 g: 0.37) and it decreased between the equal-density and the big set, *t*(14) = 2.47, *p* = 0.013, *d* = 0.64 (60 g: 0.06, 140 g: 0.002). Please note that here the difference for 230 g is automatically zero.

According to the model, the extent to which the density estimate influences heaviness perception depends on its reliability. Here, this should be reflected in a Visibility x Density Set interaction. Indeed, Density Set-related effects were further modified by Visibility, interaction Visibility × Density Set, *F*(2.83,39.6) = 43.3, *p* < 0.001, *η*_*p*_^*2*^ = 0.76. As predicted, the influence of density increased with increasing Visibility (see also S1 Fig). For the linear contrasts, we first computed the differences in heaviness estimates between two density sets for every Visibility condition (averaged over all Mass conditions). Subsequently, we computed a linear contrast over all Visibility conditions. The average difference between the small and the big set increased with Visibility, *F*(1,14) = 94.8, *p* < 0.001, *η*_*p*_^*2*^ = 0.87 (no vision: 0.04, poor: 0.35, medium: 0.49, full: 0.58). This also holds for the differences between the small and the equal density set, *F*(1,14) = 61.4, *p* < 0.001, *η*_*p*_^*2*^ = 0.81 (no vision: 0.06, poor: 0.34, medium: 0.46, full: 0.52) and the differences between the equal density and the big set, *F*(1,14) = 11.9, *p* = 0.002, *η*_*p*_^*2*^ = 0.46 (no vision: -0.02, poor: 0.01, medium: 0.04, full: 0.07). Furthermore, these differences were additionally modified by Mass, interaction Visibility × Density Set × Mass, *F*(5.53,77.4) = 5.4, *p* < 0.001, *η*_*p*_^*2*^ = 0.28. We computed linear contrasts over pairwise differences between two density sets over all Mass and Visibility conditions. For all Density Set combinations, the differences in heaviness estimates due to Mass increased with Visibility. This was true for the differences of small versus equal-density, *F*(1,14) = 18.0, *p* < 0.001, *η*_*p*_^*2*^ = 0.56, equal-density versus big, *F*(1,14) = 26.0, *p* < 0.001, *η*_*p*_^*2*^ = 0.65, and for the differences of small versus big set, *F*(1,14) = 3.2, *p* = 0.047, *η*_*p*_^*2*^ = 0.19. No other effects were observed in the ANOVA (*p* > 0.2).

A crucial prediction of our model is that objects of the same density should be perceived as more similar with increasing reliability of the density estimate. Then, the weight assigned to the mass estimate should decrease and mass information should contribute less to the final percept. In our data, this should be reflected in smaller differences within objects from the equal-density set with increasing Visibility. Average differences between successive stimuli in the equal-density set (139 versus 55 g and 230 versus 139 g) were 0.78 and 0.71 for the blindfolded condition, 0.63 and 0.65 in the poor, 0.58 and 0.59 in the medium as well as 0.63 and 0.62 in the full vision condition. A linear contrast over all Mass and Visibility conditions showed that these differences decreased with Visibility, *F*(1,14) = 3.41, *p* = 0.043, *η*_*p*_^*2*^ = 0.20. A trial-wise analysis of the visual size-weight illusion can be found in the supplementary material (S3 Fig). We did not find evidence that the illusion changed over the course of one visibility condition.

#### Model fit

We fit the cue integration model (Eqs 4 and 7) to the heaviness estimates averaged over participants (Fig 2). This allows to compare the observed heaviness estimates against model predictions. The model had seven free parameters. *a* and *x* describe the postulated relation between physical mass and the mass estimate as a power function. *b* and *y* are the corresponding counterparts for the density estimate. The three weights *w*_poor_, *w*_medium_ and *w*_full_ refer to the relative contribution of the density estimate to the overall heaviness estimate in each visibility condition. *w*_novis_ for the no vision condition was set to 0. Note that estimates derived from mass correspondingly contribute with 1–*w*, because weights sum up to 1. Also note that the four conditions were fitted simultaneously, with the same *a*, *x*, *b*, and *y* in all conditions. The fit explained 98% of variance in the data. Fitted values were *a* = 0.006, *x* = 1.06, *b* = 1.27, and *y* = 0.537. Weights increased, as expected, with visibility and were *w*_poor_ = 0.14, *w*_medium_ = 0.21 and *w*_full_ = 0.29. Heaviness estimates predicted from the model fit are included in Fig 2 as lines.

**Fig 3:**
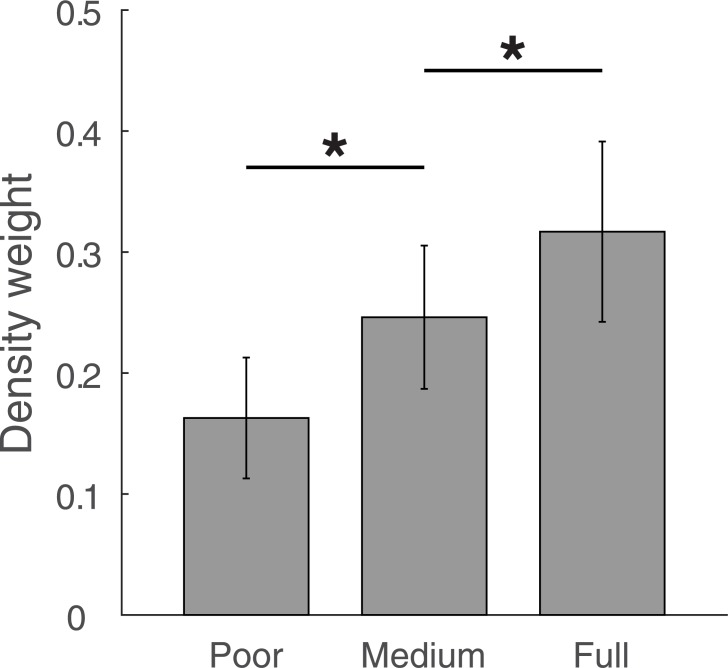
Density weights in Experiment 1. Fitted density weights for the poor, the medium and the full vision condition. Weights are averaged over individual participants with error bars denoting the 95% confidence interval of between-participant variability. * p < 0.05.

We additionally fit the model to each individual data set. The model explained more than 85% variance per individual (median: 94.4%). Individually fitted parameters (S2 Fig) spread around the parameters for the aggregated data model fit (*a*: 0.0007–0.0271, *x*: 0.75–1.51, *b*: 0.59–2.1, *y*: 0.27–2.06; *w*_poor_: 0.01–0.38; *w*_medium_: 0.02–0.47; *w*_full_: 0.03–0.57) with few outliers. For example, 14 out of 15 values for the *y* parameter lie between 0.27 and 0.77, with the remaining one (2.06) being an outlier. In 14 out of 15 cases, weights of single individuals were larger for medium as compared to poor vision, *t*(14) = 4.3, *p* < 0.001, *d* = 1.11, and they were larger for full as compared to medium vision in 13 out of 15 cases, *t*(14) = 3.7, *p =* 0.001, *d* = 0.96. Fig 3 depicts density weights.

For the no vision condition, density weights were set to 0. If they were included as additional parameter, this would have resulted in a weight of *w*_novis_ = 0.017 (range: 0–0.082, median: 0.008) without affecting other parameters much (changes < 3% for each parameter). Due to the fact that the *x* parameter is close to 1, heaviness estimates in the blindfolded condition show an almost perfect linear relation with physical mass, *r* = 0.997, *p* < 0.001, *R*^*2*^ = 0.994. This also highlights that participants perceive mass only in the blindfolded condition.

### Discussion

The model predicts that the perceived difference between objects from the equal-density set depends on mass only. In case of the big- and the small-volume set higher mass coincides with higher density. This should lead to larger differences in perceived heaviness between objects of different mass. In line with this prediction, perceived heaviness increased the least with mass for the equal-density and the most for the small-volume set, where differences in mass coincide with largest differences in density. This indicates that absolute density values contribute to perceived heaviness in addition to the contribution of object mass.

Further, with increasing visibility, density effects on perceived heaviness increased. These effects were e.g. larger differences between density sets. At the same time, the effects of mass information decreased: Objects which differ in mass but are constant in density (equal-density set) were judged as more similar with increasing visibility. We conclude that a better visibility is associated with a higher reliability of the density estimate and the latter determines the contribution of density and weight information on perceived heaviness.

The noise correlation could be affected by the noise in the estimates of mass and density. In this case, it might not be constant across conditions, but it may vary, for example depending on the properties of the currently lifted object and the way that it is lifted. Higher values of noise correlation could be obtained when the density estimates’ noise is mostly determined by the noise of the mass estimate and not so much by other noise sources (e.g. noise in the size estimate). This would for example be the case (i) under conditions with reliable size information and (ii) for small-sized objects, because uncertainty decreases with lower perceived stimulus intensity (Weber’s law). Thus, the noise correlation should (i) increase with visibility and (ii) it should be larger for small set objects. A higher noise correlation would lead to a decrease of the weight given to the less reliable cue (Eq 5), i.e. to the density estimate and would predict a decrease in the illusion magnitude with increasing visibility, especially for small objects. This is the opposite from what can be observed in the data and suggests that the noise correlation parameter can either be assumed to be relatively constant across conditions or that any influence of the error correlation parameter due to the lifting condition are negligibly small compared to the influence of absolute density values and the relative reliabilities.

There is only a single observation that seems not to fit the predictions: Differences between the small and big set were not largest for the highest mass (Fig 2). We speculate that this is might be due to anchoring [49], specifically due to a tendency of participants to stay at least approximately within the range of magnitudes reported in the first condition. All participants started with the blindfolded condition, but in the following conditions small set objects should be perceived as heavier. Consequently, for the heaviest small set stimulus, participants would have to report magnitude estimates that are considerably higher than any of the preceding ones, which they might have avoided.

Finally, we directly fit the weighted-average model to the heaviness judgments. The model explained almost all variance both in the individual (> 85%, median: 94.4%) and the average data (98%). The weights for heaviness estimates derived from density systematically increased with visibility, with 0.14 in the poor, 0.21 in the medium and 0.29 in the full vision condition. Weights shifted in the same manner with visibility for each single individual. Taken together, the results both from the fit and from inference statistics strongly support our model of heaviness perception that perceived heaviness is a weighted average of one estimate derived from mass and another estimate derived from density with weights that follow the estimates relative reliability.

## Experiment 2: The haptic size-weight illusion (magnitude estimation)

If the SWI is caused by the integration of a mass and a density estimate, and if differences in illusion strength can be explained by different relative reliabilities of the two estimates, then it should not matter whether size is perceived haptically or visually. With this experiment we aim to show that the pure haptic size-weight illusion also depends on mass, density and the relative reliability of each estimate. Therefore, the illusion strength should not only vary as a function of visibility (Experiment 1), but also as a function of the haptic reliability with which size and, thus, density can be perceived.

Rather than artificially manipulating the quality of haptic volume information, we wish to make use of the naturally occuring differences between different grip types and haptic perception: When participants have to identify an object’s shape, they choose to enclose the object with their hand(s). Such enclosure leads to better size discrimination compared to other exploratory procedures [50]. Thus, enclosure can be considered a grip type which should provide a reliable signal about object size. Enclosure involves object contact with a large skin surface area and thus many sensory fibers. A precision grip on the other hand, where the object is touched and held with thumb and forefinger, involves less skin contact and less sensory fibers. This might lead to a less reliable signal on an object’s shape and size. The least reliable signal is achieved when no haptic information about size is available, i.e. when blindfolded participants lift objects via a string.

In Experiment 2 we used the same stimulus sets and tested the same predictions as in Experiment 1. But instead of manipulating the reliability of the density estimate via visibility, participants were constantly blindfolded and we aimed to manipulate the reliability of density estimates by letting participants lift objects with different grip types (string vs. precision vs. enclosure). We tested the assumption that size information from enclosure is more reliable than size information from a precision grip with a subset of participants. Grip types which differ in their quality regarding haptic size information should also differ in the reliability of the information they provide about density. Moreover, we modified the instructions to prevent participants from staying in the range of values reported in the first condition. Instead of instructing them to "keep their subjective heaviness scale constant", they were told to "judge every single object relative to all previously lifted objects" and were reminded to do so with the start of every new lifting condition.

### Methods

#### Participants

20 healthy and naïve students from Giessen University took part for course credit (mean age = 24 years, range = 19–32, 10 female, 19 right-handed, 1 left-handed according to self-report). 10 of them had previously taken part in Experiments 3 as well as in the size discrimination task. As we found no difference between these groups, we pooled the data.

#### Setup, stimuli, design and procedure

We used the same setup and a subset of the stimuli described in Experiment 1 (60, 140 and 230 g for the small and big set each; 55, 139, 165 and 198 g for the equal-density set). All objects had screw caps which were replaceable and there were two screw caps for each object, a plain one and one with a string and bead attached (Fig 1A).

The design comprised three within-subject variables: Mass (60, 140, 230 g), Density set (small set, big set, equal-density set) and Grip Type (lifting with string, precision grip, enclosure). The 230 g stimulus from the big set was also included in the analysis of the equal-density set as it had the same density of 0.39 g/cm^3^. 55 and 139 g stimuli from the equal-density set were matched with the 60 and 140 g stimuli from the volume sets. The 165 and 198 g stimulus were not considered for the ANOVA but considered for the model fit. We included these two additional objects to prevent objects from being recognized due to their size or weight and their heaviness judged from memory.

Instructions, trial procedure and judgments were mostly equivalent to Experiment 1. In contrast to Experiment 1, participants were constantly blindfolded throughout the experiment. Instructions on how to lift the objects were given right before each grip type condition, together with the remark to "judge the heaviness of every single object relative to all previous objects". In the precision and enclosure condition, participants were instructed to pre-shape their hand for a precision grip or an enclosure. The experimenter then placed the stimulus on the felt pad and moved the participants’ right hand towards the stimulus so that their right thumb touched the object. In the enclosure condition, participants were additionally instructed to haptically explore the object for 2–3 seconds before lifting.

Lifting conditions were presented in a fixed order and every participant started with the string condition before proceeding to the precision grip and enclosure condition. This was done to prevent the potential transfer of information into conditions of lower reliability. After initial instructions, every participant ran a practice block in the string condition to get familiar with the procedure and build up a subjective heaviness scale. In the practice block, every stimulus was lifted once in a random order. Practice trials were not considered in the analysis. The experiment comprised 180 trials per participant (10 stimuli × 3 lifting conditions × 6 blocks) and lasted approximately 1.5 hours including 2 breaks of 3–5 minutes.

#### Size discrimination task

Before the proper experiment a subset of 10 participants completed a size discrimination task. These were the same participants as in Experiments 3. In this task, participants were blindfolded and had to grip objects of a small or big volume set either with a precision grip or enclose it with the full hand. We used the method of constant stimuli and a two interval forced choice paradigm (2IFC). Thus, we repeatedly presented one out of eight comparison stimuli together with the standard stimulus. Comparison stimuli systematically varied in volume and participants had to indicate which of the two was larger. For every comparison, we converted binary responses into “proportion larger than standard” and fitted a cumulative Gaussian to the data using MLE methods in psignifit 2.5.6 [51]. The just-noticeable difference (JND) is the difference in size which is required to distinguish a comparison stimulus from the standard in a certain number of trials. Thus, a lower JND is indicative of a better discrimination performance. We assessed the JND for size by the standard deviation of the underlying Gaussian. This corresponds to the difference between the size which is judged heavier in 84.1% of trials and the size which is judged heavier half of the time. The squared inverse of the JND is proportional to the reliability of a signal and thus lower JNDs indicate higher reliabilities.

We used two sets of cylindrical volume stimuli made of gray plastic. The standard stimuli had the same volume as the stimuli for the heaviness discrimination task (31.8 and 596 cm^3^). The comparisons varied in steps of 5% from 80 to 120% of the standard’s volume. Volumes were 25.4, 27, 28.6, 30.2, 33.3, 34.9, 36.5 and 38.1 cm^3^ for the small and 476.8, 506.6 536.4, 566.2, 625.8, 655.6, 685.4 and 715.2 cm^3^ for the big set. The ratio of height to diameter was 1.2 for both sets.

In every trial, participants had to grip and compare two stimuli with regard to their volume. The experimenter placed either the standard or a comparison at the felt pad and guided the participants’ right thumb towards the object. Participants were instructed to touch the object for about 2–3 seconds. To prevent the weight information from influencing size judgments [52], participants were not allowed to lift or move the stimuli. This was controlled by the experimenter. Then they removed their hand and waited for the experimenter to place down the second object. After each trial, participants had to indicate which of the two objects was larger. The order of comparison and standard was counterbalanced. Half of the participants started with the precision grip condition and the small set, the other half with a precision grip and the big set. This was done for 10 blocks. In every block, all comparisons were presented once in a random order. Subsequently, participants performed 10 blocks on the other volume set before proceeding to the enclosure condition. This sequence was repeated so that every participant completed 20 blocks for every combination of Grip Type and Set. The task consisted of 640 trials per participant (8 comparisons × 20 blocks/repetitions × 2 Sets × 2 Grip Types) and lasted approximately 3–4 hours.

As we collected data from the same participants, recording sessions were interleaved with sessions from Experiments 3. Data from different experiments were recorded in separate sessions. The order of the two experiments was balanced across participants and within each experiment, every participant completed 10 blocks in every condition. The same individual order of sessions was then repeated for the size-discrimination task. The whole recording lasted between 6–8 hours and was split into sessions of approximately 2 hours with short breaks every 15–20 minutes. Afterwards, participants completed the magnitude estimation experiment on another day.

### Results

#### Size discrimination task

A grip type which provides a more reliable size percept, should lead to a more reliable density estimate and thus to a stronger size-weight illusion. We measured just-noticeable differences (JNDs) in size discrimination for two different volume sets, a small and a big one, and two different grip types, precision grip and enclosure. To compare JNDs across both sets, we converted JNDs into proportion of the standard stimulus (Weber fraction). In the precision grip condition, Weber fractions were 0.18 and 0.11 for the small and the big set, respectively. When enclosing the object, Weber fractions were 0.14 and 0.10. For the small set, participants were, as predicted, significantly better in judging the objects size in the enclosure condition, *t*(9) = 2.81, *p* = 0.01, *d* = 0.89 (one-tailed). No significant benefit was found for the big set, *t*(9) = 0.49, *p* = 0.32, *d* = 0.15.

#### Heaviness estimates

Individual heaviness estimates were entered into a repeated-measures ANOVA comprising the variables Density Set (big, equal-density, small set), Mass (60, 140, 230 g) and Grip Type (lifting with string, precision grip, enclosure). We defined the order of Grip Type conditions as the order of their assumed reliability of the density estimate (string, precision grip, enclosure). As in Experiment 1, p-values and degrees of freedom from the ANOVA were corrected according to Greenhouse-Geiser if appropriate and we examined single aspects of our hypotheses by applying planned comparisons (t-tests and linear contrasts, all one-tailed). The analysis was completely equivalent to Experiment 1, where it is described in more detail. The analyses supported all model predictions.

**Fig 4:**
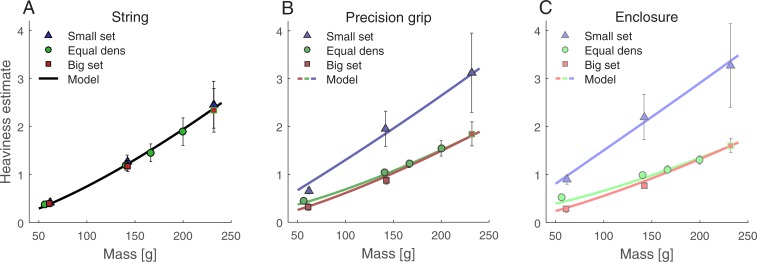
Main results of Experiment 2. Heaviness estimates averaged over individual heaviness scores with error bars denoting 95% confidence intervals of between- participant variability and lines being the model fit. (A, B, C): Heaviness estimates for different density sets as a function of mass with a different panel for every grip type (A: string, B: precision grip, C: enclosure).

As expected, heaviness estimates for the 60, 140 and 230 g stimuli, on average, significantly increased with Mass (Fig 4, see also S1 Fig), main effect Mass, *F*(1.03,19.6) = 64.3, *p* < 0.001, *η*_*p*_^*2*^ = 0.77 (60g: 0.48, 140g: 1.27 and 230 g: 2.27). They were significantly smaller for 60 g than for 140 g, *t*(19) = 11.1, *p* < 0.001, *d* = 2.48, which in turn were smaller than for 230 g objects, *t*(19) = 6.6, *p* < 0.001, *d* = 1.47. Heaviness estimates also, on average, significantly differed between the Density Sets, main effect Density Set, *F*(1.01,19.2) = 27.6, *p* < 0.001, *η*_*p*_^*2*^ = 0.59 (big set: 1.07, equal-density set: 1.15, small set: 1.81). Overall, estimates for the big set were smaller than estimates for the equal-density set, *t*(19) = 6.6, *p* < 0.001, *d* = 1.48, which in turn were smaller than estimates for the small set, *t*(19) = 5.0, *p* < 0.001, *d* = 1.11. Thus, as predicted heaviness estimates systematically increased with increasing density.

Higher differences in physical density should lead to higher differences in perceived heaviness. In our stimuli, the absolute density difference between two sets depends on its mass (Fig 1B). The effect of density should thus be modulated by mass. This is reflected by the Density Set × Mass interaction, *F*(1.17,22.3) = 13.2, *p* = 0.001, *η*_*p*_^*2*^ = 0.41. As in Experiment 1, linear contrasts confirmed that differences between the small and the big set increased with increasing mass, *F*(1,19) = 11.4, *p* = 0.002, *η*_*p*_^*2*^ = 0.37 (60 g: 0.33, 140 g: 0.87, 230 g: 1.02). The same is true for differences between the small and the equal-density set, *F*(1,19) = 15.2, *p* < 0.001, *η*_*p*_^*2*^ = 0.44 (60 g: 0.21, 140 g: 0.74, 230 g: 1.02). For the equal-density and the big set, we did not find evidence that differences in heaviness estimates decreased with increasing mass, *t*(19) = -0.73, *p* = 0.726, *d* = -0.16 (60 g: 0.12, 140 g: 0.13). In total, the differences between heaviness estimates reflected the differences in physical density.

The difference between density sets should perceptually become more pronounced, the more reliable the density estimate. This is reflected in the Density Set × Grip Type interaction, *F*(1.15,21.8) = 22.5, *p* < 0.001, *η*_*p*_^*2*^ = 0.54. As revealed by linear contrasts (cf. Exp. 1), the difference between the small and the big set increased with Grip Type, *F(*1,19) = 27.7, *p* < 0.001, *η*_*p*_^*2*^ = 0.59 (string: 0.08, precision: 0.9, enclosure: 1.23) The same is true for the differences between the small and the equal-density set, *F*(1,19) = 20.9, *p* < 0.001, *η*_*p*_^*2*^ = 0.52 (string: 0.08, precision: 0.81, enclosure: 1.08), and the differences between the big and the equal-density set, *F*(1,19) = 44.1, *p* < 0.001, *η*_*p*_^*2*^ = 0.70 (string: 0.001, precision: 0.01, enclosure: 0.15).

The way density and mass information interact was further modulated by Grip Type, interaction Mass × Grip Type, *F*(1.58,30.0) = 3.6, *p* = 0.049, *η*_*p*_^*2*^ = 0.41, interaction Mass × Density Set × Grip Type, *F*(1.56,29.6) = 11.9, *p* < 0.001, *η*_*p*_^*2*^ = 0.39. For all Density Set combinations, the differences in heaviness estimates due to Mass increased with Grip Type, as revealed by linear contrasts (cf. Exp. 1). This was true for small versus big set, *F*(1,19) = 10.7, *p* = 0.002, *η*_*p*_^*2*^ = 0.36, small versus equal density set, *F*(1,19) = 17.4, *p* < 0.001, *η*_*p*_^*2*^ = 0.48, as well as for the differences between equal-density and big set, *F*(1,19) = 91.6, *p* < 0.001, *η*_*p*_^*2*^ = 0.83.

We performed a separate analysis of the equal-density set to test a critical prediction of our model: due to the integration of density information, objects of the same density should be perceived as more similar with increasing reliability of the density estimate. This should be reflected in heaviness estimates of the equal-density set becoming more similar with a grip type providing more reliable information about density (Fig 4, see also S1 Fig). Average differences between subsequent stimuli (140 versus 60 g and 230 versus 140 g) were 0.81 and 1.14 for the string condition, 0.6 and 0.81 for the precision grip condition and 0.46 and 0.62 for the enclosure condition. Differences significantly decreased with Grip Type, *F*(1,19) = 18.7, *p* < 0.001, *η*_*p*_^*2*^ = 0.5. A trial-wise analysis of the haptic size-weight illusion can be found in the supplementary material (S4 Fig). We did not find evidence that the illusion changed over the course of one grip type condition.

#### Model fit

We fit the cue integration model (Eqs. 4 and 7) to the heaviness estimates averaged over participants (Fig 4). The model had six free parameters: *a*, *b*, *x* and *y*, *w*_precision_ and *w*_enclosure_. The latter two denote the relative contribution of the density estimate to the combined heaviness estimate in the corresponding Grip Type condition. The fit explained 99.4% of variance in the data. Fitted values were *a* = 0.0014, *x* = 1.37, *b* = 1.08, and *y* = 0.74. Weights increased, as expected, with haptic reliability and were *w*_precision_ = 0.31 and *w*_enclosure_ = 0.42. Heaviness estimates predicted from the model fit are included as lines in Fig 4.

**Fig 5:**
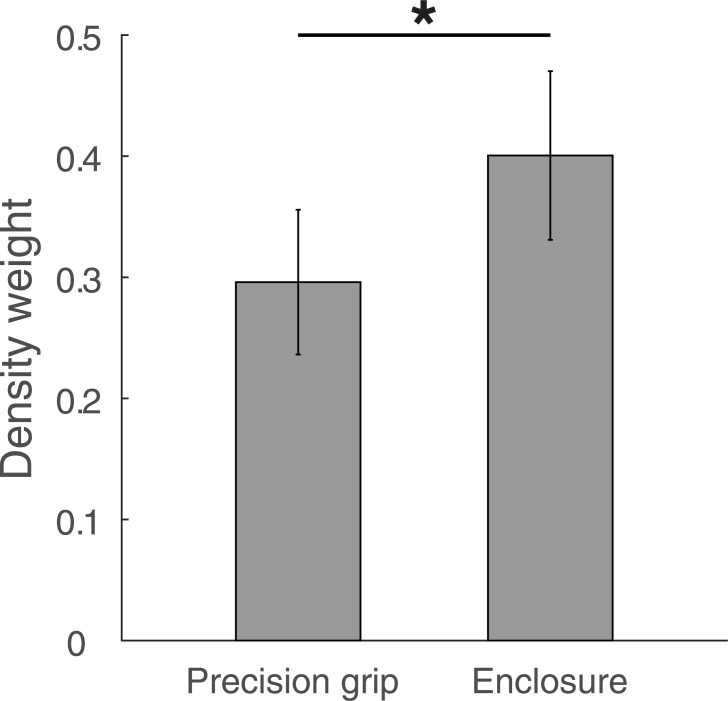
Density weights in Experiment 2. Fitted density weights for the precision grip and the enclosure condition. Weights are averaged over individual participants with error bars denoting the 95% confidence interval of between-participant variability.

In addition, we fit the model to each individual data set. The model explained more than 93% variance per individual (median: 97.3%). Ranges for individually fitted parameters (S2 Fig) are *a*: 0.00001–0.026, *x*: 0.76–2.4, *b*: 0.17–1.99, *y*: 0.30–2.14; *w*_precision_: 0.11–0.57; *w*_enclosure_: 0.1–0.68. As in Experiment 1, they were all spread around the parameters for the aggregated data model fit with few outliers. For example, 17 out of 20 values for the *b* parameter lie between 0.83 and 1.19. Fig 5 depicts individual weights in the precision grip and the enclosure condition. In 16 out of 20 cases, weights of single individuals were larger for enclosure compared to a precision grip and a one-tailed *t*-test confirmed that weights increase with Grip Type, *t*(19) = 4.37, *p* < 0.001, *d* = 0.98. Like in Experiment 1, the inclusion of an additional weight parameter for the string condition (*w*_string_ = 0.03) would not have affected other parameters much (all changes < 2%). In the string condition, physical mass and perceived heaviness correlated almost perfectly, *r* = 0.994, *p* < 0.001, *R*^*2*^ = 0.99 (Fig 4A).

### Model fits across experiments

We also checked whether the model can consistently explain the data from Experiment 1 and 2 when using the same set of parameters: If the derived parameters (*a*, *x*, *b*, *y*) from Experiment 1 were used and only the density weights were fit to the aggregated data (2 free parameters), the model explained 91.8% of variance (*w*_precision_ = 0.37 and *w*_enclosure_ = 0.54). For Experiment 1, if we only fit weights and use the other parameters from Experiment 2 (3 free parameters), the model explained 80% of variance (*w*_poor_ = 0.13, *w*_medium_ = 0.2 and *w*_full_ = 0.28). If we fit the model to both data sets simultaneously (9 free parameters), the parameters were: *a* = 0.0047, *x* = 1.12, *b* = 1.20, *y* = 0.66, *w*_poor_ = 0.12, *w*_medium_ = 0.19, *w*_full_ = 0.23, *w*_precision_ = 0.35 and *w*_enclosure_ = 0.47. The fit explained 96.4% of variance. Overall, the model provides a good fit in all cases, even when parameters are exchanged across experiments.

### Discussion

In the size discrimination experiment, we tested whether enclosing an object indeed leads to a better size discrimination performance compared to touching it with a precision grip. As predicted, Weber fractions for small objects were lower in the enclosure compared to the precision grip condition, suggesting more reliable size estimates during enclosure. We failed to show this for big objects.

In the magnitude estimation experiment, blindfolded participants lifted objects either on a string or with one of the two grip types that were also tested in the size-discrimination task. Observed heaviness estimates were fully consistent with model predictions: high-density objects were judged heavier than objects with the same weight but lower density. Within and between sets, differences in perceived heaviness increased with increasing differences in density. All density-related effects were further modified by the way objects were lifted. Density effects were absent when objects were lifted on a string and thus no information about size was available. They were present, however, whenever participants lifted the objects with a grip that provided information about the objects size. Density-related effects were largest when objects were lifted with a grip type (enclosure) which was shown to go along with more reliable size estimates-at least for small objects. Moreover, the model fit to the heaviness judgments explained almost all variance (99.4%) and density weights were highest in the enclosure condition. Interestingly, and consistent with previous findings [28,30], the average density weight for the full strength haptic illusion (0.42) was higher than the weight for the full visual illusion obtained in Experiment 1 (0.29). Across experiments, parameters were in similar ranges (S2 Fig), and showed only little deviations regarding the common parameters (*a*, *x*, *b*, *y*). The model was also well able to fit the data from both experiments simultaneously with shared parameters. This corroborates the notion that the visual and haptic size-weight illusion share the same mechanism and emphasizes that our model is able to account for both of them.

Like in Experiment 1, the data do not support the notion that the noise correlation parameter varied substantially across the experiment. Such a dynamic change would have decreased the density contribution especially in the enclosure condition (see Experiment 1 for discussion). This is the opposite from what can be observed in the data. We take this as evidence that also in Experiment 2, the influence of the error correlation parameter was negligibly small compared to the influence of density and the relative reliability.

Taken together, we conclude that a more reliable haptic estimate of the object’s size leads to a more precise, i.e. more reliable, density estimate and that this reliability determines the contribution of density to the combined heaviness percept.

## Experiment 3: The haptic size-weight illusion (2IFC)

The two previous experiments have demonstrated that our model can well describe heaviness perception as a function of density and mass using the method of magnitude estimation. This method is well-established when investigating perception, but at the same time it is also clear that higher-level, cognitive processes beyond perception may bias magnitude estimates [53,54] (e.g. tendencies to report multiples of 5 and 10, to stick to values previously reported or to exaggerate). Hence, to put our conclusions on even more solid grounds, Experiment 3 tested model predictions using psychophysical methods that are less prone to be influenced by cognitive processes and compared the results to the individual model parameters obtained by magnitude estimation. A subset of participants from Experiment 2 took part in Experiment 3. This experiment assessed the strength of the haptic size-weight illusions when lifting objects with a precision grip or with full enclosure using a two-interval forced choice (2IFC) task. In every trial, blindfolded participants had to decide which of two stimuli felt heavier: the standard stimulus or a comparison. Standard and comparisons either both belonged to the small or to the big set used in Experiments 1 and 2. Comparison stimuli were lifted on a string and thus should be judged according to their mass only (Experiment 1 and 2). Standard stimuli were either lifted with a precision or a full enclosure of the hand and participants should thus susceptible to the illusion when lifting standard stimuli (Experiment 2). Consequently, the point of subjective (PSE) for the standard stimulus compared to the comparison stimuli is informative about the magnitude of the illusion. The PSE is defined by the mass which is judged heavier (and lighter) than the standard stimulus at a rate of 50%. We assessed the standards’ point of subjective equality (PSE) as compared to the comparison stimuli. We predicted that due to the integration of density information, the standards’ PSE values should differ from their physical mass. The difference between PSE value and physical value was predicted to be larger when objects are lifted with the hand fully enclosing the object compared to lifting it with a precision grip. Big standard objects should be perceived as lighter than they are, meaning that PSE values are below the physical mass of the standard. The opposite pattern was expected for the small set (cf. Fig 4). Additionally, we predicted individual PSE shifts from individual model parameters obtained in Experiment 2.

### Methods

#### Participants, setup and stimuli

10 healthy undergraduate students aged 20 to 32 from Giessen University (mean age: 25 years, 5 female, 1 left-handed according to self-report) took part for course credits. All participants also took part in Experiments 2.

The setup was identical to that in Experiment 1 and 2. Like in the previous experiments, stimuli were white cylindrical plastic cans with a screw cap. Stimuli either belonged to the small (*V*_small_ = 32 cm^3^) or to the big volume set (*V*_big_ = 596 cm^3^). For the small set, the mass of stimuli ranged in 11 steps from 170 to 230 g (170, 180, 186, 194, 198, 200, 202, 206, 214, 220 and 230 g) with 200 g being the standard stimulus. Thus, densities, ranged from 5.36 to 7.24 g/cm^3^. For the big set, we used 14 stimuli (11 stimuli equivalent in mass to the small set and 3 additional comparisons of 60, 100 and 140 g). Densities ranged from 0.1 to 0.39 g/cm^3^. The screw cap of standard stimuli was plain, whereas for all comparisons, a string of 20 cm length and 1.3 cm diameter was attached to its center.

#### Design and procedure

The experiment comprised two differently sized stimulus sets (Density Set: small set, big set) and two different ways of lifting the standard stimulus (Grip Type: precision grip, enclosure). For every combination of Grip Type and Density Set, we measured the point of subjective equality (PSE) using a 2IFC task.

Within each trial, blindfolded participants had to lift the standard stimulus and one comparison in a random order. Afterwards they had to indicate which of the two felt heavier. Depending on the condition, the standard stimulus was lifted with a precision grip or by fully enclosing the object, whereas all comparisons were lifted on a string. A detailed explanation of the lifting procedure is given in the methods of Experiments 1 and 2. Within each block, every comparison was lifted once in a random order. Thus, a block consisted of 10 trials for the small and 13 trials for the big set. All participants completed 10 blocks of the precision grip condition for both density sets before proceeding to the enclosure condition. Half of the participants started with the small, the other half with the big set.

To prevent fatigue and collecting uninformative data, we discarded comparison stimuli depending on the participants' responses: After the first two blocks, we discarded stimuli if more than three of the heaviest stimuli were consistently judged as heavier (or if more than three of the lightest stimuli were consistently judged as lighter). E.g. if the heaviest four comparisons were throughout judged as heavier in the first two blocks (and the fifth heaviest was not), we refrained from presenting the heaviest one for the remaining experiment. After five blocks, we discarded additional comparison stimuli, if two or more of the heaviest comparisons were always judged as heavier than the standard (or if two or more of the lightest stimuli were consistently judged as lighter). In total, the experiment could consist of up to 460 trials (small set: 2 Grip types × 10 comparisons × 10 blocks; big set: 2 Grip types × 13 comparisons × 10 blocks). On average 95 trials per participant were not presented by intention due to the aforementioned routine. In a further 37 trials the experimenter made an error and missed presenting a stimulus. This corresponds to less than four trials per participant and less than one trial for every individual condition. On average 362 trials per participant were available for analysis (range: 330–419). Data recording was finished within one or two sessions and lasted 2–3 hours. This session was interleaved with data recording for the size discrimination task in Experiment 2 (see Experiment 2, Methods, Size discrimination task).

#### Data analysis and prediction of PSE values

For every comparison stimulus, we computed the “proportion heavier than standard” responses and fitted a cumulative Gaussian to the individual data of every Density Set and Grip Type using psignifit 2.5.6 and MLE methods [51]. From every fit, we derived the point of subjective equality (PSE). PSE values were entered into a 2 × 2 ANOVA with the two within-subject factors Grip Type and Density Set. Predicted effects were further tested by planned comparisons (*t*-tests, all one-tailed).

In Experiments 1 and 2, we have shown that when objects can neither be seen nor touched, perceived heaviness depends on mass only. In this case, no density estimate can be derived and perceived heaviness is solely determined by the mass estimate. When lifting objects with a precision grip or enclosure, perception is additionally influenced by the object’s density (Exp. 2). In this case, perceived heaviness is supposed to be a weighted average of a mass and a density estimate. In the present experiment, we measure points of subjective equality (PSE), i.e. how heavy a comparison stimulus needs to be in order to be perceived as equally heavy as the standard stimulus. Comparisons were lifted using a string (mass information only) whereas standards were lifted with a grip type providing information about both mass and density. To predict individual PSE values, we therefore equated the mass estimate with the full model:

$${\hat{h}}_{m}(m)=\hat{h}(m_{s},\rho_{s})$$ (8)

with:

$${\hat{h}}_{m}(m)=a∙{PSE}_{predicted}^{x}$$ (9)

$$\hat{h}(m_{s},\rho_{s})=(1-w_{\rho })∙a∙{m_{s}}^{x}+w_{\rho }∙b∙{\rho_{s}}^{y}$$ (10)

where *m*_*s*_ and *ρ*_*s*_ are stimulus properties of the standard stimulus and *a*, *x*, *b*, *y* and *w*_*ρ*_ are individual parameters derived from Experiment 2. The predicted PSE value is given by:

$${PSE}_{predicted}=\sqrt[{x}]{\frac{\hat{h}(m_{s},\rho_{s})}{a}}$$ (11)

### Results

**Fig 6:**
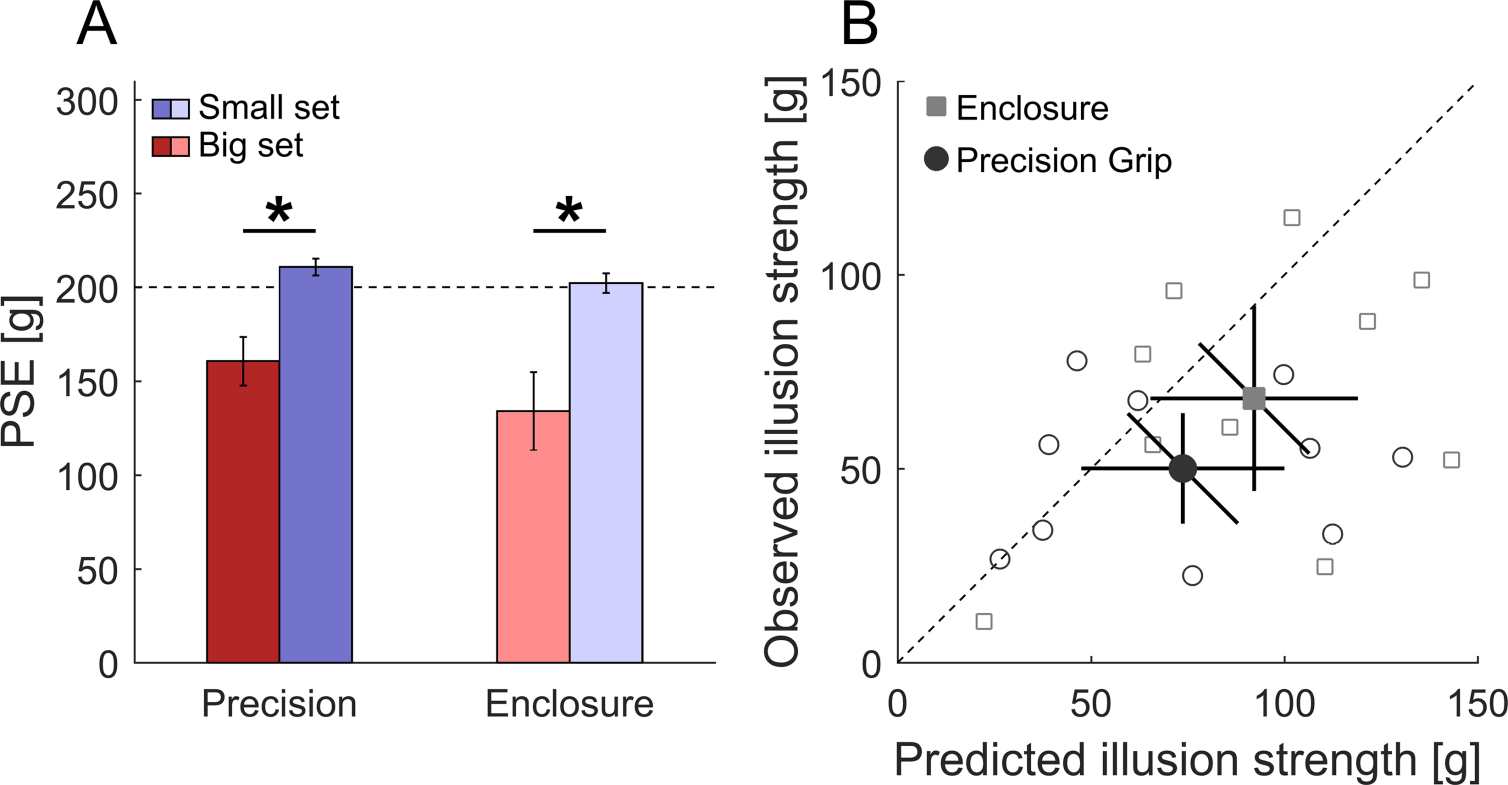
Main results of Experiment 3. (A) Average PSE values for the small (purple) and the big set (red) with 95% confidence intervals. (B) Observed against predicted illusion strength for both grip types. Lighter gray squares indicate the enclosure condition. Open symbols denote individuals. Diagonal error bars for the aggregated data mark the error of the differences between observed and predicted values and have to be compared to the identity line.

Average PSE values (Fig 6A) for the big set were 161 g (*SD* = 20 g) for the precision grip and 134 g (*SD* = 32 g) for the enclosure condition. For the small set, PSE values were 211 g (*SD* = 7 g) for the precision grip and 202 g (*SD* = 8 g) for the enclosure condition. A difference in perceived heaviness due to the SWI had been predicted to result in PSE values deviating from the stimulus’ physical mass of 200 g. For the big set, PSE values in both conditions are significantly lower than 200 g, precision grip: *t*(9) = 6.24, *p* < 0.001, *d* = 1.97, enclosure: *t*(9) = 6.53, *p* < 0.001, *d* = 2.07. For the small set, PSE values were above 200 g for the precision grip, *t*(9) = 4.91, *p* < 0.001, *d* = 1.55, but not for the enclosure condition, *t*(9) = 0.87, *p* = 0.204, *d* = 0.28.

A 2 × 2 repeated-measures ANOVA with the two within-participant factors Grip Type and Density Set revealed a main effect of Density Set, *F*(1,9) = 56.9, *p* < 0.001, *η*_*p*_^*2*^ = 0.86. Standard stimuli from the small set were judged heavier than standard stimuli from the big set, both for the precision grip, *t*(9) = 7.94, *p* < 0.001, *d* = 2.51, as well as the enclosure condition, *t*(9) = 6.45, *p* < 0.001, *d* = 2.04. This is the classical size-weight illusion. The strength of the illusion depended on the Grip Type condition, interaction Density Set × Grip Type, *F*(1,9) = 5.69, *p* = 0.041, *η*_*p*_^*2*^ = 0.39. Differences between small and big set were larger with an enclosure (*M* = 68 g, *SD* = 33 g) compared to a precision grip (*M* = 50 g, *SD* = 20 g), *t*(9) = 2.39, *p* = 0.02, *d* = 0.75. For the big set, PSE values were, as expected, lower in the enclosure compared to the precision grip condition, *t*(9) = 4.01, *p* = 0.002, *d* = 1.27. Violating our predictions, PSE values for the small set were not largest in the enclosure condition, *t*(9) = -2.60, *p* = 0.99, *d* = 0.82. As PSE values decreased with Grip Type for the big set, but did not increase with Grip Type for the small set, we also found a main effect of Grip Type, *F*(1,9) = 23.5, *p* = 0.001, *η*_*p*_^*2*^ = 0.75.

In Fig 6B we plot the observed illusion strength (difference between small and big object) as a function of the predicted illusion strength based on the model and the parameters derived from Experiment 2. The predicted illusion strength was M = 74 g (SD = 37 g) in the precision grip and M = 92 g (SD = 37 g) in the enclosure condition. For the big set, predictions were *M* = 165 g (*SD* = 19 g) for the precision grip and *M* = 155 g (*SD* = 19 g) for the enclosure condition. For the small set, we predicted *M* = 238 g (*SD* = 18 g) and *M* = 247 g (18 g).

### Discussion

The aim of the present experiment was to reassess the strength of the haptic size-weight illusion and its dependence on the grip type by using a two-interval forced choice (2IFC) task. Whereas top-down biases can influence magnitude estimation, these biases would not be expected in a 2IFC experiment [53,54]. Using magnitude estimation, we had shown in Experiment 2 that the strength of the haptic SWI is larger when lifting objects with full enclosure compared to lifting with a precision grip. In the present experiment, participants compared the heaviness of a standard stimulus lifted with a grip type providing information about its size and density (precision grip, enclosure) to the heaviness of comparison stimuli lifted on a string. We assumed that participant’s heaviness perception of the comparisons would be entirely based on object mass. The basic pattern of results was consistent with our predictions–at least as regards the big standard: Small standard objects were perceived as being heavier than big standard objects and the difference was more pronounced when participants used a full enclosure instead of a precision grip. This is the classical size-weight illusion and it is, as predicted, larger in lifting conditions when density information is supposed to be more reliable. The results corroborate our conclusion that the strength of the illusion is determined by the relative reliability of sensory size information.

Further as expected, the PSE values for the big set of objects indicated that these objects were perceived to be lighter than they physically are, namely 161 and 134 g in the precision grip and enclosure condition, respectively, as compared to the actual mass of 200 g. In contrast to our predictions, the PSE values for the small set did not unequivocally demonstrate that these objects were perceived as being heavier than they are (211 and 202 g, respectively), and they hardly fit the values predicted from the model (238 and 246 g for the small set). That is, small objects for which size information was available were perceived to be approximately the same weight as objects lifted on a string. This had not been observed in Experiment 1 and 2 and previous research [30], where small objects were perceived as being heavier than objects lifted via string.

One reason for this discrepancy between the experiments might lie in the assumption that participants based their heaviness perception entirely on the perceived mass, when they lifted objects via string. This assumption is feasible when participants lack any idea on the size of the objects, as was the case in Experiment 1 and 2 where all participants started with the string conditions. However, in Experiment 3 from start on objects were alternatingly lifted via a string or by a grip that provided information on object size. This information might have been transferred into lifting via a string and participants might have included a perceptual prior on the object’s density when assessing their weight. For example, this could be a prior that the comparison’s density is in a similar range as the small object’s density. Thus, comparisons would be perceived as heavier than they actually were and reduce observed PSE values in both sets. In line with this interpretation of the discrepancy is that PSE values not only for small objects but also for big objects (161 and 134 g) were also lower than their predicted values (165 and 155 g).

Taken together, using a 2IFC task, we replicated the finding from the magnitude experiments that the strength of the size-weight illusion increased with the reliability with which size is perceived. Moreover, the present results may suggest that the assumption that heaviness judgments are solely formed from the object's mass when lifting objects without visual or haptic size information is only true when participants are not given any idea about the object’s size (as in Experiment 1 and 2). But this assumption may not hold when alternating between lifting with size information present or absent.

## General discussion

The present study aimed to test the model that perceived heaviness is a weighted average of two heaviness estimates: One estimate is derived from the object’s mass, and the other is derived from the object’s density. Weights for each estimate are supposed to depend on the estimates’ relative reliabilities and their correlated noise. Overall, the results strongly support the notion that an object’s density biases heaviness perception to an extent that depends on the relative reliability of the density estimate.

In Experiments 1 and 2, participants had to lift objects of different masses and densities and judge their heaviness while we manipulated the reliability of the density estimate. We did not manipulate the noise correlation, because there is no feasible assumption for changes in the error correlation parameter that could explain the size-weight illusion and our results. Our results showed that the perceived difference of two equally-weighted stimuli cannot be a direct function of size. If size information directly contributed to heaviness, then the perceived difference for two equally-weighted stimuli should depend on their size only and would be independent of their mass [33]. This would have led to an additive effect between small and big set objects. However, the interaction observed in the results of both experiments suggests that density information contributes to heaviness perception: The difference in heaviness judgments for two equally-weighted objects increased with an increasing difference in density.

This notion is also consistent with the results of the equal-density set. These stimuli differed in mass but were identical in density. Hence, heaviness judgments of these stimuli are supposed to differ with respect to the mass estimate but are identical with respect to the density estimate. Thus, the stronger the illusion and the higher the weight of the density estimate, the more similar judgments of the equal-density set should be. This is exactly what we observed: The stronger the illusion, the more similar equally dense objects were judged.

### The illusion strength is modulated by reliability

In this study, we further showed that the strength of the size-weight illusion depends on the quality of size and density information–independent of the modality. Because we aimed to prevent that participants transfer any information about size/density into conditions with lower quality of information, we presented the visibility and lifting conditions in Experiment 1 and 2, respectively, in a constant order. In both experiments all participants started with a lifting condition where they were blindfolded and lifted objects on a string. Consecutively, the assumed quality of size and thus density information increased with every following condition. As a consequence, we cannot ultimately exclude the possibility that fatigue or an increased experience with the stimuli contributed to the difference across visibility/ lifting conditions. One solution to this problem would have been to use a between-participant design. However, we decided to use a within design for several reasons: First, we wished to have sufficient statistical power. Second, we had aimed to fit the model also across all lifting conditions per individual, which would not have been possible with data from a between-participant design. There are a number of good arguments why we think that differences between visibility conditions in Experiment 1and between the different grip types in Experiment 2 are better explained by information quality as compared to by order effects. Previous research showed that illusion magnitudes did not vary over repeated liftings, both for the size-weight [7,11,55] and the material-weight illusion [56]. Also in our data, we did not find evidence that the illusion magnitude systematically varied within one block (S3 and S4 Figs). In addition, Cross and Rotkin [35] also noticed that the strength of the illusion depends on the way weights are lifted. They found that in their study when objects were lifted with two hands, the illusion was stronger compared to the study by Stevens and Rubin [34] who used one-handed lifting. Taken together, this suggests that the difference between the visibility conditions in Experiment 1 and grip type conditions in Experiment 2 is not caused by fatigue or order effects.

Cross and Rotkin [35] concluded that the difference between their results and the results by Stevens and Rubin [34] is due to the maximum amount of weight that can be comfortably lifted [35]. Regarding our grip types, it is likely that more weight can be lifted when an object is enclosed with the hand compared to lifting it with a precision grip. Thus, this interpretation might explain the modulation by grip type we found in Experiment 2. However, it cannot account for the modulation of illusion strength by visibility observed in the first experiment. Here, all objects were lifted identically. Rather, our data strongly support the assumption that the strength of the size-weight illusion depends on the reliability with which density can be perceived. This assumption appears to be consistent with the data by Cross and Rotkin [35]. Compared to lifting with two fingers only (precision grip), lifting with multiple fingers (enclosure) elicited a stronger illusion. When enclosing the object with the whole hand, more skin area and more sensory fibers are involved. This should reduce noise and make the sensory signal more reliable. The same should be true for lifting with both hands compared to one-handed lifting.

### Potential influences of lifting kinematics, sway and response type

As we did not measure lifting kinematics (e.g. grip and load force) we could not control that the different visibility conditions in Experiment 1 and the different grip types used in Experiment 2 may have led to differences in the forces applied during lifting which then caused differences in perceived heaviness. Moreover, lifting kinematics in Experiment 1 may have been further affected by the fact that participants could see the experimenter handling the object. Lifting kinematics are affected by restricting vision [11] as well as observing other peoples’ lifting behavior [57]. Yet, although lifting kinematics were originally considered as the potential cause for the size-weight illusion [8], several studies have meanwhile dismissed the relationship between kinematics and the size-weight illusion [9,12] and the observation of other peoples’ lifts influences lifting kinematics without affecting perceived heaviness [57]. Thus, even if there were systematic differences in lifting kinematics they will most likely not have affected our perceptual data to a meaningful extent.

Participants were instructed to lift objects slowly using up- and down movements and the experimenter controlled the participants’ behavior, assuring, for example, that there was no lateral sway in Experiment 1 where objects were lifted on a string. Swaying the objects laterally could provide alternative information about the object’s mass and size. As we did not record the object’s position, we cannot exclude subtle lateral movements which remained unnoticed to the experimenter. However, even if there was lateral sway undetected by the experimenter, this could have only affected our results when participants had varied the lateral sway systematically from one visibility condition to the other. In the precision grip condition in Experiment 2 and 3, participants lifted objects using their thumb and index finger. Any deviation away from a central axis through the object might have provided cues to torque or inertia which is known to contribute to the size-weight illusion [37]. If inertia due to unprecise grasp points contributed to the size-weight illusion in the precision grip condition, then the illusion should be stronger in the precision grip condition. However, this is not evident in our data. Our data show that the illusion is stronger when objects are lifted with full enclosure of the hand compared to a precision grip which is consistent with the interpretation that the magnitude of the illusion depends on the quality of information.

Our model predicts that the difference in perceived heaviness between two equally-weighted objects should not only depend on their difference in size, but also on their absolute mass. Both, Experiment 1 and 2 clearly supported this notion. This argues against the averaging model by Anderson [33] which predicts that the illusion should be independent of mass. However, using another response method than we did, Anderson found no evidence that the size-weight illusion was modulated by mass. In his study he employed a rating procedure with two anchors. Participants had to rate the heaviness of objects on a scale from 1 to 20 where 1 would correspond to a light anchor (50 g) and 20 would correspond a heavy anchor (500 g). Further, a study by Sarris and Heineken [58] suggests that the modulation of the size-weight illusion by mass is dependent on the response method. They observed the modulation when using magnitude estimation but not with the rating procedure [58]. Thus, the generalizability of part of the evidence in the present study that favors our mass-density model over the mass-size model [33] may be limited by effects of the used response methods. However, a modulation of the illusion by mass has recently also been shown when participants report the perceived heaviness ratio between two stimuli [59]. Another crucial prediction of our model was replicated by using a two-interval forced choice method in Experiment 3: We showed that the illusion magnitude depends on the quality with which size information can be perceived. Thus, this phenomenon seems to be independent of the applied response method. As we only had one mass for the standard stimuli in Experiment 3 (200 g), we could not test whether the modulation of the illusion by mass can also be found with a 2IFC method. This could have been further support for our model and might have supported the notion that the illusion also depends on mass when other response methods are used.

### How can density be perceived?

A direct haptic cue to perceive density might be the pressure, i.e. the force per area that an object exerts on the skin while being held in the hand. This relates back to the early explanation of the SWI by Charpentier [1] who concluded that the sensation of heaviness decreases when the weight is distributed over a wider skin surface area. While this appears plausible at first sight, it cannot account for our data: When blindfolded participants lifted objects on a string, they touched the wooden bead with index finger and thumb. In this case, participants were not deceived by the illusion. They were however, when stimuli in Experiment 2 were lifted with a precision grip. In both scenarios, the skin surface area is approximately equal. Moreover, it cannot explain the visual size-weight illusion where weight information is perceived by the haptic and size information by the visual modality.

A recent study suggested a model assuming that in case of two lifted objects, the brain evaluates all categorical density relationships between objects and from that infers the final weight estimate [59]. This model can explain several findings on the size-weight illusion for two simultaneously lifted objects and highlights that priors driving heaviness perception might be related to the density of an object. The model does not refer to magnitude estimates of heaviness but to the perceived heaviness ratio of two objects and it predicts that the illusion magnitude depends on the volume relationship and the assumed density relationship of both objects [59]. However, it falls short in explaining why the illusion magnitude depends on mass which has been shown in the literature [34–36] and is also mirrored in the findings of our first two Experiments. The dependence of the illusion magnitude by mass does not only emerge when using magnitude estimates but also when the illusion is reported as a ratio of two heaviness percepts as becomes evident from the authors own data [59].

The results by Chouinard et al. [42] provide instructive insights to the perception of density. Using an fMRI adaptation paradigm, they identified the brain regions which code for weight, size and density: The primary motor area (M1) adapted to the weight of objects. And whereas size was coded in sensory areas in the parietal and temporal cortex, density was coded in a higher order area, the ventral premotor area (PMv). This may suggest that an amodal representation of object density is derived from sensory information about weight and size. Such a density representation (or density estimate) would be able to explain why seen as well as felt size can induce the illusion and bias our judgment of heaviness. While haptic information about weight and size can directly be compared, felt weight and seen size first need to be transformed into the same sense-specific or amodal code before a density estimate can be derived. Transformations are known to increase the variability of a signal [60,61] and this additional transformation in case of the visual illusion might thus decrease the reliability of the density estimate and lead to a lower density weight compared to the haptic SWI. It was suggested that it would be optimal for the brain to minimize the number of transformational stages [61]. This might also explain why visual size information does not incrementally contribute to the illusion [30] when both, visual and haptic information about size are available and the brain seems to make use of haptic size information only.

### Other weight illusions and expectations

The size-weight illusion is by far the strongest of all weight illusions and there is reason to assume that other weight illusions involve different mechanisms and might actually arise due to expectations. Another famous weight illusion is the material-weight illusion [55,62,63]. This illusion describes the phenomenon that among two equally-weighted and equally-sized objects with different surface materials, the object which appears to be made out of a denser material is perceived as lighter. This weight illusion is perfectly in line with the idea that current sensory input is contrasted with prior expectations. In accordance with that, its magnitude does usually not exceed half of the size-weight illusion magnitude [7,64] and thus coincides with the magnitude of a size-weight illusion that is induced by expectation alone [11]. Moreover, it occurs independently of the size-weight illusion, because the SWI is not modulated by the apparent material [7,65]. Although our current model cannot account for other weight illusions, it would be possible to extend the model by adding an additional estimate. Nevertheless, it remains questionable whether this would be necessary to sufficiently describe heaviness perception in everyday life, because the material-weight illusion requires the violation of apparent material and the actual object density which is not given under normal lifting conditions. Even if this manipulation is applied, the much weaker influence of apparent material might cause too little additional variability to justify the inclusion of additional parameters when heaviness perception is measured over a broad range of masses, sizes and also apparent materials.

### The size-weight illusions is a density-mass illusion

As gravity is more or less constant on our planet, an object’s mass and its weight are proportional and the two words are often used interchangeably. However, as has been shown by Plaisier and Smeets [66], gravitational forces are not necessary for the illusion to occur; it can also be induced by inertial forces. They concluded that the illusion is caused by the object’s mass, not by its weight. Still, this leaves us with the question why the brain should, in addition to information about mass, integrate information about density for the perceptual judgment of heaviness? One explanation might be that it is more efficient to store information about density rather than mass (or weight) in visuomotor memory. Then, the memory of density together with a visual size-analysis would be sufficient to predict its weight and form an appropriate motor command [43,44]. This way, one visuomotor representation would be sufficient to generate adequate motor commands for a whole family of objects made from the same material.

Using information about density would help us to differentiate between materials, because then the brain integrates that piece of information which is constant for a given material: its density. And as we know from the well-studied visual modality, the brain is an expert in identifying objects and their properties although the sensory input might be changing (e.g. size-constancy, color-constancy). By emphasizing the component which is a property of the material, rather than the object, the density-mass illusion appears to be the haptic and visuo-haptic contribution to create constancy in a world where sensory information is varying throughout.

## Supporting information

S1 Fig Heaviness estimates for the different density sets and lifting conditions. Same data as in Fig 2 and Fig 4 but with a separate panel for data of each density stimulus set (A, B: big set; C, D: equal density set; E, F: small set). Left column (A, C, E) are data from Experiment 1, the right column are data from Experiment 2. The black and gray lines represent the mass and the density estimate. Whereas the mass estimate is identical within one experiment (column), the density estimate is also different for every density set.
(EPS)

S2 Fig Distribution of parameters for the model fits in Experiment 1 and 2. Open black circles denote values for single individuals, red squares parameters for the model fit to the averaged data. Panels depict parameters *a* (A), *b* (B), *x* (C), *y* (D) and the density weights in experiment 1 (E) and 2 (F). For the meaning of parameters, refer to Eqs 4 and 5.
(EPS)

S3 Fig Repetition effects visual illusion. Trial-wise heaviness estimates from Experiment 1 for the 60 g (top row), 140 g (middle row) and 230 g stimuli (bottom row) of the small set (blueish/purple) and the big set (reddish). Error bars are 95% confidence intervals of between-participant variability and horizontal lines are model predictions. To test whether the visual size-weight illusion is influenced by repetition, we conducted a repeated-measures ANOVA with the four factors mass (60, 140, 230), set (small, big), visibility (no vision, poor, medium, full) and trial within block (1 to 6). Any set × trial interaction would indicate that the size-weight illusion is affected by repetition and that the difference between visibility conditions might be influenced by order effects. The ANOVA revealed no trial × set interaction, *F*(5,70) = 0.56, *p* = 0.73, *η*_*p*_^*2*^ = 0.039, no trial × set × mass interaction, *F*(10,140) = 0.25, *p* = 0.99, *η*_*p*_^*2*^ = 0.017, no trial × set × visibility interaction, *F*(15,210) = 1.12, p = 0.34, *η*_*p*_^*2*^ = 0.074 and no trial × set × mass × visibility interaction, *F*(30,420) = 1.21, *p* = 0.21, *η*_*p*_^*2*^ = 0.08. Thus, we did not find evidence that the illusion varied over the time of one block which suggests that the difference between visibility conditions was not caused by order effects or fatigue.
(EPS)

S4 Fig Repetition effects haptic illusion. Trial-wise heaviness estimates from Experiment 2 for the 60 g (top row), 140 g (middle row) and 230 g stimuli (bottom row) of the small set (blueish/purple) and the big set (reddish). Error bars are 95% confidence intervals of between-participant variability and horizontal lines are model predictions. To test whether the haptic size-weight illusion is influenced by repetition, we conducted a repeated-measures ANOVA with the four factors mass (60, 140, 230), set (small, big), grip type (string, precision, enclosure) and trial within block (1 to 6). Any set × trial interaction would indicate that the size-weight illusion is affected by repetition and that the difference between grip type conditions might be influenced by order effects. The ANOVA revealed no trial × set interaction, *F*(3.22,61.19) = 0.99, *p* = 0.41, *η*_*p*_^*2*^ = 0.049, no trial × set × mass interaction, *F*(3.14,59.69) = 0.84, *p* = 0.48, *η*_*p*_^*2*^ = 0.042, no trial × set × grip type, *F*(2.57,48.75) = 1.11, *p* = 0.35, *η*_*p*_^*2*^ = 0.055, and no trial × set × mass × grip type interaction, *F*(20,380) = 1.48, *p* = 0.084, *η*_*p*_^*2*^ = 0.072. Thus, we did not find evidence that the illusion varied over the time of one block which suggests that the difference between grip type conditions was not caused by order effects or fatigue.
(EPS)
